# Supplementary material for: Splice-Junction-Based Mapping of Alternative Isoforms in the Human Proteome
Source: Cell Rep. Author manuscript; Available in PMC 2020 Jan 15. (PMC6961840; doi:10.1016/j.celrep.2019.11.026)
Supplement: 2 [file NIHMS1546469-supplement-2.zip › DF1/PXD006675/VentricularSeptum/VentricularSeptum_24_TPM1_AISEELDHALNDMTSGPMLK.pdf]

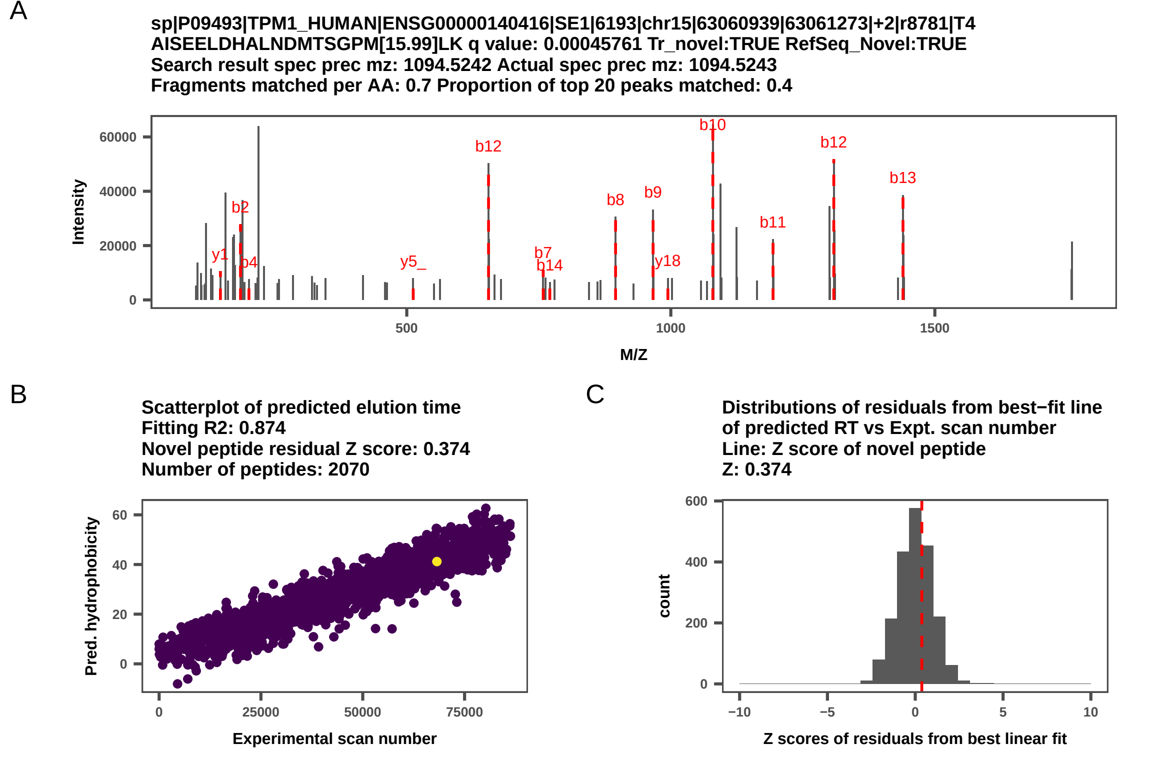
A
sp|P09493|TPM1_HUMAN|ENSG00000140416|SE1|6193|chr15|63060939|63061273|+2|r8781|T4
AISEELDHALNDMTSGPM[15.99]LK q value: 0.00045761 Tr_novel:TRUE RefSeq_Novel:TRUE
Search result spec prec mz: 1094.5242 Actual spec prec mz: 1094.5243
Fragments matched per AA: 0.7 Proportion of top 20 peaks matched: 0.4
Intensity
M/Z
y1
b2
b4
y5_
b12
b7
b14
b8
b9
y18
b10
b11
b12
b13
B
Scatterplot of predicted elution time
Fitting R2: 0.874
Novel peptide residual Z score: 0.374
Number of peptides: 2070
Pred. hydrophobicity
Experimental scan number
C
Distributions of residuals from best−fit line
of predicted RT vs Expt. scan number
Line: Z score of novel peptide
Z: 0.374
count
Z scores of residuals from best linear fit
